# Association Between Prescription Opioid Therapy for Noncancer Pain and Hepatitis C Virus Seroconversion

**DOI:** 10.1001/jamanetworkopen.2021.43050

**Published:** 2022-01-12

**Authors:** James Wilton, Stanley Wong, Roy Purssell, Younathan Abdia, Mei Chong, Mohammad Ehsanul Karim, Aaron MacInnes, Sofia R. Bartlett, Rob F. Balshaw, Tara Gomes, Amanda Yu, Maria Alvarez, Richard C. Dart, Mel Krajden, Jane A. Buxton, Naveed Z. Janjua

**Affiliations:** 1 British Columbia Centre for Disease Control, Vancouver, British Columbia, Canada; 2 Department of Emergency Medicine, University of British Columbia, Vancouver, British Columbia, Canada; 3 School of Population and Public Health, University of British Columbia, Vancouver, British Columbia, Canada; 4 Centre for Health Evaluation & Outcome Sciences, St Paul's Hospital Vancouver, British Columbia, Canada; 5 Pain Management Clinic, Jim Pattison Outpatient Care & Surgical Centre, Fraser Health Authority, Surrey, British Columbia, Canada; 6 Department of Anesthesiology, Pharmacology & Therapeutics, Faculty of Medicine, University of British Columbia, Vancouver, British Columbia, Canada; 7 Department of Pathology and Laboratory Medicine, University of British Columbia, Vancouver, British Columbia, Canada; 8 Kirby Institute, University of New South Wales, Sydney, New South Wales, Australia; 9 George and Fay Yee Centre for Healthcare Innovation, University of Manitoba, Winnipeg, Manitoba, Canada; 10 Leslie Dan Faculty of Pharmacy, University of Toronto, Toronto, Ontario, Canada; 11 Li Ka Shing Knowledge Institute, St Michael's Hospital, Toronto, Ontario, Canada; 12 ICES, Toronto, Ontario, Canada; 13 Rocky Mountain Poison and Drug Safety, Denver Health and Hospital Authority, Denver, Colorado; 14 Department of Emergency Medicine, University of Colorado Health Sciences Center, Denver

## Abstract

**Importance:**

Initiation of injection drug use may be more frequent among people dispensed prescription opioid therapy for noncancer pain, potentially increasing the risk of hepatitis C virus (HCV) acquisition.

**Objective:**

To assess the association between medically dispensed long-term prescription opioid therapy for noncancer pain and HCV seroconversion among individuals who were initially injection drug use–naive.

**Design, Setting, and Participants:**

A population-based, retrospective cohort study of individuals tested for HCV in British Columbia, Canada, with linkage to outpatient pharmacy dispensations, was conducted. Individuals with an initial HCV-negative test result followed by 1 additional test between January 1, 2000, and December 31, 2017, and who had no history of substance use at baseline (first HCV-negative test), were included. Participants were followed up from baseline to the last HCV-negative test or estimated date of seroconversion (midpoint between HCV-positive and the preceding HCV-negative test).

**Exposures:**

Episodes of prescription opioid use for noncancer pain were defined as acute (<90 days) or long-term (≥90 days). Prescription opioid exposure status (long-term vs prescription opioid–naive/acute) was treated as time-varying in survival analyses. In secondary analyses, long-term exposure was stratified by intensity of use (chronic vs. episodic) and by average daily dose in morphine equivalents (MEQ).

**Main Outcomes and Measures:**

Multivariable Cox regression models were used to assess the association between time-varying prescription opioid status and HCV seroconversion.

**Results:**

A total of 382 478 individuals who had more than 1 HCV test were included, of whom more than half were female (224 373 [58.7%]), born before 1974 (201 944 [52.8%]), and younger than 35 years at baseline (196 298 [53.9%]). Participants were followed up for 2 057 668 person-years and 1947 HCV seroconversions occurred. Of the participants, 41 755 people (10.9%) were exposed to long-term prescription opioid therapy at baseline or during follow-up. The HCV seroconversion rate per 1000 person-years was 0.8 among the individuals who were prescription opioid–naive/acute (1489 of 1947 [76.5%] seroconversions; 0.4% seroconverted within 5 years) and 2.1 with long-term prescription opioid therapy (458 of 1947 [23.5%] seroconversions; 1.1% seroconverted within 5 years). In multivariable analysis, exposure to long-term prescription opioid therapy was associated with a 3.2-fold (95% CI, 2.9-3.6) higher risk of HCV seroconversion (vs prescription opioid-naive/acute). In separate Cox models, long-term chronic use was associated with a 4.7-fold higher risk of HCV seroconversion (vs naive/acute use 95% CI, 3.9-5.8), and long-term higher-dose use (≥90 MEQ) was associated with a 5.1-fold higher risk (vs naive/acute use 95% CI, 3.7-7.1).

**Conclusions and Relevance:**

In this cohort study of people with more than 1 HCV test, long-term prescription opioid therapy for noncancer pain was associated with a higher risk of HCV seroconversion among individuals who were injection drug use–naive at baseline or at prescription opioid initiation. These results suggest injection drug use initiation risk is higher among people dispensed long-term therapy and may be useful for informing approaches to identify and prevent HCV infection. These findings should not be used to justify abrupt discontinuation of long-term therapy, which could increase risk of harms.

## Key Points

**Question:**

Is long-term prescription opioid therapy for noncancer pain associated with hepatitis C virus (HCV) seroconversion among individuals who initially had no history of injection drug use?

**Findings:**

In this cohort study of 382 478 individuals who had a baseline negative HCV test followed by at least 1 additional test, long-term prescription opioid therapy among those who were initially injection drug use–naive was associated with a statistically significant 3.2-fold higher risk of HCV seroconversion vs no long-term therapy. Overall, approximately 1% of exposed individuals seroconverted within 5 years.

**Meaning:**

The findings of this study suggest that the risk of injection drug use initiation may be higher among people dispensed long-term prescription opioid therapy, leading to increased HCV seroconversion risk.

## Teaser

This cohort study examines the incidence of hepatitis C seroconversion in individuals prescribed opioid therapy for noncancer pain.

## Introduction

Despite a steady decrease in hepatitis C virus (HCV) diagnoses through the 1990s and 2000s in many high-income countries, there has been a resurgence in recent years. In the US, the estimated annual number of acute HCV diagnoses increased by 280% between 2010 and 2017 (from 11 800 to 44 700).^1^ There is also evidence of a smaller increase in Canada (approximately 50% increase in the rate between 2011 and 2017 among people aged 20-40 years).^2^ Injection of drugs is currently the main route of HCV acquisition and recent changes in HCV rates may be due to increases in the number of people who inject drugs.^3,4^

Since the early 2000s, the use of prescription opioids to manage pain in North America has increased substantially.^5^ Canada and the US have consistently been 2 of the highest consumers of prescription opioids globally over the past 2 decades,^5^ despite evidence suggesting that prescription opioids provide limited benefit in managing chronic noncancer pain.^6,7^ Long-term prescription opioid therapy is also associated with a dose-dependent risk of adverse outcomes, including dependence and overdose.^6,8^ In recent years, several policies and interventions have been introduced to reduce prescription opioid–related harms, but evidence of their effectiveness is limited.^9^

Initiation of injection drug use may be more frequent among people with a history of medically dispensed prescription opioid therapy for pain,^10^ potentially leading to an increased risk of HCV acquisition if drug use equipment is shared. The association between self-reported nonmedical prescription opioid use (prescription opioids primarily obtained from sources other than a physician) and initiation of heroin, a commonly injected drug, is well established,^11,12^ although the overall rate of transition appears to be infrequent (3%-4% of individuals initiate heroin within 5 years of nonmedical use of prescription opioids).^13^ Less is known about medically dispensed prescription opioids,^10,14^ although a recent cohort study found long-term prescription opioid therapy for pain to be associated with initiation of injection drug use.^10^ Although these associations may not be causal, qualitative studies suggest that some people attribute their initiation of heroin/injection drug use to prior medical or nonmedical prescription opioid use.^15,16,17,18,19^ In particular, sudden discontinuation of prescription opioid therapy, potentially owing to more stringent policies and guidelines, may lead some individuals to initiate heroin/injection drug use and inadvertently increase the risk of harm.^18,20^ There is also concern that the introduction of tamper-resistant formulations may have had a similar effect.^21^ Epidemiologic changes in HCV in parts of the US can be characterized by the emergence of a cohort of young people (aged <30 years) who inject drugs and often report a history of oral nonmedical prescription opioid use prior to injection drug use initiation.^22,23,24^

To date, few studies have directly assessed the association between medically dispensed prescription opioid therapy for pain and HCV seroconversion among individuals who initially have no history of injection drug use (injection drug use–naïve). An ecological study has implicated prescription opioid policies in increasing HCV diagnoses in the US^21^ and 2 administrative case-control analyses have identified prior dispensation of prescription opioids as a risk factor for HCV diagnosis.^25,26^ However, these studies assessed HCV diagnosis, and most analyses of HCV seroconversion have focused on the association between nonmedical prescription opioid injection and HCV acquisition among people who already inject drugs.^27,28^ In this present analysis, we examined the association between medically dispensed prescription opioid therapy for noncancer pain and HCV seroconversion among individuals who were initially injection drug use–naive in a large population-based cohort of people who had more than 1 HCV test.^29^

## Methods

### Data Sources

We used the IDEAs data platform (also known as the British Columbia Hepatitis Testers Cohort).^29^ The databases integrated within the IDEAs platform and their linkage have been described (eTable 1 in the Supplement).^29^ In brief, IDEAs includes all individuals tested for HCV at the British Columbia Centre for Disease Control Public Health Laboratory (BCCDC-PHL) between 1992 and 2015. The BCCDC-PHL performs more than 95% of all HCV testing in the province. These data are linked to information on medical visits (1990-2015), hospitalizations (1985-2015), emergency department visits (2012-2015), cancers (1923-2015), pharmacy dispensations (1996-2015), deaths (1985-2015), and HCV laboratory testing (1992-2017).

Reporting of this analysis followed the Strengthening the Reporting of Observational Studies in Epidemiology (STROBE) reporting guideline for cohort studies. This study was reviewed and approved by the University of British Columbia Research Ethics Board. Informed consent is not required for secondary analysis of administrative deidentified data.

### Study Population

We limited our analysis to individuals with at least 2 HCV test results, of which the first was HCV antibody-negative, to identify HCV seroconversions and estimate the date of HCV seroconversion and time at-risk of acquisition. Other analyses of retrospective cohorts have also used this approach to identify risk factors for HCV, HIV, and other infectious diseases.^30^

In this study, we included all individuals in the BCCDC-PHL database whose first HCV antibody test result between January 1, 2000, and December 31, 2015, was HCV-negative. Given this inclusion criterion, we expected most incident seroconversions to be acquired through injection drug use rather than unsafe medical procedures (eg, contaminated blood donations, transfusion-transmitted infections).

We excluded individuals with (1) no follow-up HCV test (antibody, polymerase chain reaction, or genotype) before December 31, 2017, (2) a first HCV antibody–negative test conducted when the participant was younger than 18 years or older than 65 years, or (3) a history of substance use, opioid agonist therapy, or HIV infection prior to the first HCV-negative test (to limit analysis to individuals who were initially injection drug use–naive).

A history of a substance use problem was identified using diagnostic codes from hospitalization, medical visit, and ambulatory care records (eTable 2 in the Supplement). Opioid agonist therapy was identified through pharmacy dispensation records and a fee item code from medical visits. In a validation study using the IDEAs data platform and linked surveillance data with self-reported injection drug use, these substance use and opioid agonist therapy codes had high sensitivity (91%) but lower specificity (72%) in identifying injection drug use (only validated for individuals aged 11-65 years; excluded alcohol use).^31^ To further increase sensitivity, we excluded individuals with a history of an alcohol use problem or who were living with HIV.

### Incident HCV Seroconversion 

Seroconversion was defined as a positive HCV antibody, polymerase chain reaction, or genotype test following a negative HCV antibody test. The midpoint between the negative and positive test results was used to approximate the date of seroconversion.^32^

### Prescription Opioid Therapy 

Our main exposure of interest was long-term prescription opioid therapy for noncancer pain. Data on prescription opioid medications were obtained from PharmaNet, a provincewide database capturing dispensations from all community pharmacies, regardless of payer, from 1996 onward.

We used an episode approach to assess patterns of prescription opioid use among participants.^33,34^ An episode starts with an incident prescription opioid dispensation and ends once there have been 6 months with no drug supply. Each episode has 3 distinct but related measures: episode length (number of days between episode start and episode end, includes gaps in drug supply), days of drug supply (number of calendar days within an episode covered by drug supply), and episode intensity (percentage of episode days covered by drug supply). These measures were used to categorize episodes as acute or long-term (episodic or chronic) (Table 1).^33,34,35^ A previous study assessed the face validity of these definitions and provided additional rationale for their selection.^34^ In building episodes of prescription opioid use, low-dose codeine formulations (<30 mg per tablet), prescription opioid formulations primarily used for cough suppression or opioid agonist therapy, injectable prescription opioid formulations, and prescription opioid dispensations occurring after a cancer/palliative care record were not considered.^34,35^

**Table 1.** Prescription Opioid Episode Definitions

Episode type	Definition^a^
Acute	<90 episode days
Long-term	≥90 episode days
Episodic	<90 d of drug supply and/or <50% episode intensity
Chronic	≥90 d of drug supply and ≥50% episode intensity

^a^ Episode days indicates number of days between episode start and episode end (includes gaps in drug supply). Days of drug supply indicates the number of calendar days within episode that were covered by drug supply. Episode intensity indicates the percent of episode days covered by drug supply.

In this analysis, an individual’s prescription opioid exposure status was treated as time-varying based on episodes initiated between 1996 and 2015. We classified prescription opioid exposure status as either unexposed (prescription opioid-naive or acute use) or exposed (long-term prescription opioid therapy). At baseline (first HCV-negative test), an individual was considered exposed only if they had an ongoing long-term episode. During follow-up, baseline prescription opioid status could change from unexposed to exposed if an individual initiated a long-term episode. Status could not change from exposed to unexposed. In secondary analyses, long-term exposure was stratified by intensity of use (episodic vs chronic) (Table 1) and by average daily dose (<90 morphine equivalents [MEQ] vs ≥90 MEQ).

We focused on long-term prescription opioid therapy as our exposure of primary interest because research suggests it is associated with limited benefit and increased risk. Prescription opioids are generally not recommended as first-line treatment for chronic noncancer pain.^36,37^ We believed that combining prescription opioid-naive and acute-exposed individuals was justified because a previous analysis found that most (70%) acute episodes contain only 7 or fewer days’ supply of opioids.^34^

### Covariate Measurement

We measured individual-level covariates covering different sociodemographic factors (sex, birth year, race, age, material/social deprivation, and regional health authority) and comorbidities (major mental illness and chronic pain). Covariates were assessed using linked data sets. Classification of race used a name recognition algorithm. This algorithm could lead to individuals who are not White to be misclassified as White because they have anglicized names, but the algorithm has high specificity for Asian names; therefore, 3 categories were used: East Asian, South Asian, and other. More information on covariate definitions can be found in eTable 2 in the Supplement.

### Statistical Analysis

We followed up eligible individuals from the date of their first HCV-negative test (baseline) to the date of HCV seroconversion or last HCV-negative test. If their last HCV-negative test or estimated date of HCV seroconversion occurred after 2015, follow-up was censored at the end of 2015. Individuals who initiated a long-term prescription opioid episode during follow-up but had developed a substance use problem between baseline and initiation of prescription opioid therapy were censored at the date of episode initiation to limit analysis to individuals who were injection drug use–naive at initiation of a long-term prescription opioid episode.

We created pseudocumulative incidence curves by applying the clock reset approach to unexposed individuals initiating long-term prescription opioid therapy after the start of the study.^38^ Multivariable Cox regression models were used to quantify the association between time-varying prescription opioid status and HCV seroconversion.^39^ All covariates in the model (except for sex and race) were treated as time-varying. Time-varying covariates were measured at baseline and, if applicable, updated at initiation of a long-term prescription opioid episode. We allowed for a 90-day lag to identify substance use problems (exclusion criteria) to account for potential delays in diagnosis. Cox regression models were stratified by age and calendar year of study start owing to violation of the nonproportionality assumption. With 2-sided, unpaired testing, significance was set at *P* < .05. All analyses were conducted using SAS/STAT software, version 9.4 (SAS Institute Inc).

## Results

Between 2000 and 2015, a total of 1 153 433 individuals tested negative on an HCV antibody test. We excluded 655 959 individuals who did not have a follow-up test before the end of 2017; 52 411 whose first HCV-negative test was conducted when they were younger than 18 years or older than 65 years; 61 918 with a history of a substance use problem (including alcohol), opioid agonist therapy, or HIV; and 667 who were missing information on sex or geography.

The final study population included 382 478 individuals, of whom more than half were female (224 373 [58.7%] vs 158 105 [41.3%] male), born before 1974 (201 944 [52.8%]), and younger than 35 years at baseline (196 298 [53.9%]) (Table 2). Mean (SD) age was 35.6 (12.3) years. These individuals contributed 2 057 668 person-years of follow-up (Table 3). Median follow-up was 4.3 (IQR, 1.9-8.3) years and individuals received a median of 2 (IQR, 2-3) HCV tests.

**Table 2.** Characteristics of Study Participants^a^

Characteristic	Baseline (N = 382 478), No. (%)	Prescription opioid–naive/acute (n = 370 889), No. (%)	Long-term prescription opioid exposure (n = 41 755), No. (%)	
			Episodic (n = 34 681)	Chronic (n = 7074)
Sex				
Male	158 105 (41.3)	153 361 (41.4)	13 851 (39.9)	3123 (44.2)
Female	224 373 (58.7)	217 528 (58.7)	20 830 (60.1)	3951 (55.9)
Calendar year				
2000-2003	98 768 (25.8)	95 062 (25.6)	6365 (18.4)	1621 (22.9)
2004-2008	120 006 (31.4)	116 409 (31.4)	12 010 (34.6)	2313 (32.7)
2009-2015	163 704 (42.8)	159 418 (43.0)	16 306 (47.0)	3140 (44.4)
Birth year				
<1965	117 361 (30.7)	110 598 (29.8)	14 616 (42.1)	4281 (60.5)
1965-1974	84 583 (22.1)	82 194 (22.2)	8333 (24.0)	1475 (20.9)
≥1975	180 534 (47.2)	178 097 (48.0)	11 732 (33.8)	1318 (18.6)
Age, y				
<25	83 543 (21.8)	82 549 (22.3)	3956 (11.4)	300 (4.2)
25-34	112 755 (32.1)	120 639 (32.5)	9212 (26.6)	1199 (17.0)
35-44	81 230 (21.2)	78 408 (21.1)	8617 (24.9)	1812 (25.6)
45-54	56 299 (14.7)	53 220 (14.4)	7371 (21.3)	2008 (28.4)
55-65	38 651 (10.1)	36 073 (9.7)	5525 (15.9)	1755 (24.8)
Race^b^				
East Asian	47 635 (12.5)	47 350 (12.8)	1469 (4.2)	120 (1.7)
South Asian	35 071 (9.2)	34 083 (9.2)	3610 (10.4)	400 (5.7)
Other	299 772 (78.4)	289 456 (78.0)	29 602 (85.4)	6554 (92.7)
Social deprivation^c^				
1 (least deprived)	71 650 (18.7)	69 854 (18.8)	6047 (17.4)	1055 (14.9)
5 (most deprived)	98 418 (25.7)	95 105 (25.6)	9335 (26.9)	2096 (29.6)
Missing	2207 (0.6)	2140 (0.6)	166 (0.5)	35 (0.5)
Material deprivation^c^				
1 (least deprived)	89 042 (10.5)	87 317 (23.5)	6083 (17.5)	1058 (15.0)
5 (most deprived)	71 883 (18.8)	68 974 (18.6)	8012 (23.1)	1724 (24.4)
Missing	2207 (0.6)	2140 (0.6)	166 (0.5)	35 (0.5)
Chronic pain	90 255 (23.6)	83 442 (22.5)	15 954 (46.0)	4403 (62.2)
Major mental health illness	37 164 (9.7)	34 641 (9.3)	6695 (19.3)	1833 (25.9)

^a^ All covariates were considered time-varying except sex, ethnicity, and birth year. Time-varying covariates were measured at either baseline (first hepatitis C virus–negative test) or start of long-term prescription opioid episode.

^b^ Race was determined using a validated name recognition algorithm (eTable 2 in the Supplement). Other includes all races or ethnicities other than those identified as East Asian or South Asian. This includes White individuals and people with anglicized names.

^c^ All levels of material/social deprivation not shown for ease of presentation. Missing material/social deprivation was retained as it may be a proxy for homelessness.

**Table 3.** Number of Participants, Follow-up Time, and Rate of HCV Seroconversion

Population	Participants, No.	Follow-up time, person-years		HCV seroconversions, No.	HCV incidence per 1000 person-years	Cumulative probability of HCV within 5 y, %^a^
		Total	Median (IQR)			
All participants	382 478	2 057 668	4.3 (1.9-8.3)	1947	0.9	0.5
Prescription opioid–naive/acute	370 899	1 837 491	3.9 (1.7-7.5)	1489	0.8	0.4
Long-term	41 755	220 178	4.5 (2.0-7.9)	458	2.1	1.1
Intensity of long-term use						
Episodic	34 681	181 862	4.4 (2.0-7.9)	347	1.9	1.0
Chronic	7074	38 315	4.6 (2.1-8.1)	111	2.9	1.6
Average daily dose for long-term use, MEQ^b^						
<90	39 763	208 919	4.4 (2.0-7.9)	420	2.0	1.1
≥90	1992	11 259	5.1 (2.2-8.6)	38	3.4	1.8

Abbreviations: HCV, hepatitis C virus; MEQ, morphine equivalent.

^a^ Cumulative probability calculated from cumulative incidence curves.

^b^ Average daily dose was calculated by dividing the cumulative MEQ during the episode by the number of episode days covered by drug supply (ie, did not consider gaps in use).

### Baseline and Follow-up Prescription Opioid Status

Most participants were classified as prescription opioid–naive at study start (370 889 [97.0%]), with the remainder having an ongoing episodic (7713 [2.0%]) or chronic (3876 [1.0%]) episode at baseline. A total of 30 166 individuals who were prescription opioid–naive/acute at baseline initiated a long-term prescription opioid episode during follow-up. Most of these long-term episodes were episodic (26 968 [89.4%]) and the remainder were chronic (3198 [10.6%]). A further 15 468 individuals began a long-term episode during follow-up but had developed a substance use problem between baseline and initiation of therapy and were therefore censored at initiation of therapy. Individuals who initiated long-term therapy (41 755) were more likely to be older, of other race, have greater social or material deprivation, and have a history of chronic pain and major mental illness (Table 2).

###  Prescription Opioid Episode-Level Characteristics

Characteristics of the first long-term episode initiated by study participants are described in eTable 3 in the Supplement. The median episode length was 228 days, with median 40 days’ drug supply, and 22.5 MEQ median daily dose for long-term episodes, and these values were higher for chronic vs episodic long-term episodes (median episode length, 1968 vs 191 days; median days’ drug supply, 1389 vs 28 days; and median dose 35.1 vs 21.2 MEQ).

### HCV Seroconversion

There were a total of 1947 HCV seroconversions during follow-up, yielding an overall incidence rate of 0.9 seroconversions per 1000 person-years (Table 3). Of individuals who seroconverted, 1456 (74.8%) had evidence of transitioning to injection drug use after the start of the study vs 24 572 (6.5%) individuals who did not seroconvert. The median time between the HCV-positive test and preceding HCV-negative test was 2.2 (IQR, 1.0-4.5) years.

Most HCV seroconversions (1489 [76.5%]) were among prescription opioid–naive/acute individuals, with the remainder among individuals exposed to long-term therapy (458 [23.5%]) (Table 3). The rate of HCV seroconversion was higher for the long-term exposed cohort (2.1 per 1000 person-years; 1.1% seroconverted within 5 years) compared with prescription opioid–naive/acute cohort (0.8 per 1000 person-years; 0.4% seroconverted within 5 years) (Table 3 and Figure). The HCV seroconversion rate was greater with more intense long-term use (chronic vs episodic) and at higher average daily doses (≥90 vs <90 MEQ).

**Figure.**
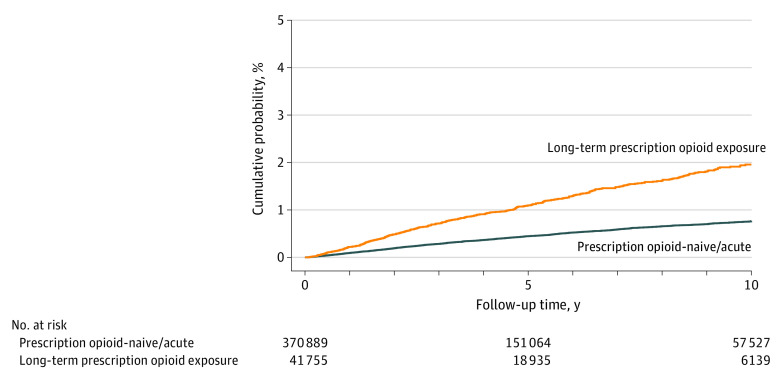
Cumulative Incidence of Hepatitis C Virus Seroconversion by Prescription Opioid (PO) Exposure Category Clock reset procedure applied to individuals initiating long-term prescription opioid therapy during follow-up. Time zero represents baseline (first HCV-negative test) or initiation of prescription opioid therapy (if initiated during follow-up).

### Multivariable Models

In the main multivariable Cox regression model, long-term prescription opioid therapy was associated with a 3.2-fold higher risk (95% CI, 2.9-3.6) of HCV seroconversion vs prescription opioid–naive/acute (Table 4). Other characteristics associated with a higher risk of seroconversion included male sex and greater material deprivation (eTable 4 in the Supplement). Characteristics associated with lower HCV risk included chronic pain and East and South Asian race vs other race. In separate Cox regression models, long-term chronic use was associated with a 4.7-fold higher risk of HCV seroconversion vs naive/acute use (95% CI, 3.9-5.8) and in another model long-term higher-dose use (≥90 MEQ) was associated with a 5.1-fold higher risk vs naive/acute use (95% CI, 3.7-7.1) (Table 4).

**Table 4.** Association Between Long-term Prescription Opioid Therapy for Noncancer Pain and HCV Seroconversion in Bivariable and Multivariable Cox Models^a^

Prescription opioid status vs prescription opioid–naive/acute	HR (95% CI)	
	Unadjusted	Adjusted
**Model 1 (main model)**		
Long-term, overall	2.9 (2.6-3.2)	3.2 (2.9-3.6)
**Model 2**		
Long-term, intensity of use		
Episodic	2.7 (2.4-3.0)	2.9 (2.6-3.3)
Chronic	3.9 (3.2-4.7)	4.7 (3.9-5.8)
**Model 3**		
Long-term, average daily dose		
<90 MEQ	2.8 (2.5-3.1)	3.1 (2.8-3.5)
≥90 MEQ	4.5 (3.3-6.3)	5.1 (3.7-7.1)

Abbreviations: HCV, hepatitis C virus; HR, hazard ratio; MEQ, morphine equivalent.

^a^ Multivariable models adjusted for sex, race, material deprivation, major mental illness, chronic pain, and local health authority. Multivariable models were stratified by calendar year (2000-2003, 2004-2008, and 2009-2015) and age (<25, 25-44, 45-54, ≥55 years) owing to violation of nonproportionality assumption. All covariates except sex and ethnicity were time-varying (measured at baseline and updated at initiation of long-term prescription opioid therapy, if applicable). Average daily dose was calculated by dividing the cumulative MEQ during the episode by the number of episode days covered by drug supply (ie, did not consider gaps in use).

## Discussion

In, to our knowledge, one of the largest cohorts of individuals tested for HCV in the world, use of long-term prescription opioid therapy for noncancer pain was associated with a higher risk of HCV seroconversion among individuals who had more than 1 HCV test and who were injection drug use–naive at baseline or initiation of therapy. Overall, the rate of HCV seroconversion among people dispensed long-term prescription opioid therapy was infrequent (2.1 HCV seroconversions per 1000 person-years; 1.1% seroconverted in 5 years). However, seroconversion risk was 3.2-fold higher with long-term vs no long-term prescription opioid therapy and there was evidence of higher risk with more intense long-term use (chronic vs episodic) and at higher daily doses (≥90 vs <90 MEQ). Our findings support 2 recent case-control analyses identifying an association between medically dispensed prescription opioids and HCV diagnosis.^25,26^ One of these analyses found that older individuals (age >40 years) with a history of prescription opioid use were 11 times more likely to test positive on an HCV antibody test,^26^ and the other analysis found prior prescription opioid dispensation to be the second most important variable for predicting HCV diagnosis in a machine learning prediction model.^25^ Our analysis builds on these studies by using a longitudinal approach with HCV seroconversion as the outcome and by distinguishing between prescription opioid dispensations for pain vs opioid agonist therapy.

Our analysis suggests that injection drug use initiation may be more frequent among people dispensed long-term prescription opioid therapy and that this may lead to a higher risk of HCV acquisition. This finding supports the well-established association between nonmedical prescription opioid use and heroin,^12^ and a recent cohort analysis identifying an association between medically dispensed long-term prescription opioid therapy and initiation of injection drug use.^10^ Although we were unable to establish a causal relationship between prescription opioids and HCV seroconversion or formally assess the role of injection drug use initiation as a mediator, several reasons or motivations for transitioning from prescription opioids to heroin or injection drug use (eg, rising tolerance, relief of withdrawal or pain due to discontinuation of therapy, and introduction of tamper-resistant formulations) have been identified in qualitative studies.^15,16,17,18,19^ To evaluate our hypothesis that injection drug use initiation risk is higher among people dispensed prescription opioids, we used an administrative algorithm with high sensitivity for identifying injection drug use^31^ to limit our analysis to individuals who were injection drug use–naive at prescription opioid initiation. Furthermore, we expected most HCV seroconversions during follow-up to be related to injection drug use because screening of blood products for HCV was implemented in the early 1990s and our analysis was limited to people with an HCV-negative antibody test in 2000 or later. We found that three-quarters of individuals who seroconverted had evidence of injection drug use after the start of the study, highlighting the role of injection drug use transition in HCV seroconversion.

Prescription opioid therapy may also be a proxy for injection drug use or HCV diagnosis/acquisition. People receiving prescription opioid therapy can have complex medical and sociodemographic profiles (eg, a higher prevalence of substance use and mental health issues),^34,40^ potentially leading to an increased baseline risk of injection drug use initiation and residual confounding. Confounding by indication is another potential issue, as uncontrolled chronic pain may lead to injection drug use initiation in and of itself.^11,12^ Other mechanisms could explain the association between prescription opioids and HCV: people with a history of prescription opioid dependence prior to injection drug use initiation tend to have higher-risk injection practices^41^ and episodic prescription opioid therapy may be indicative of an inability to access a constant supply of prescription opioids to manage pain (potentially leading to illicit drug use). Furthermore, although we attempted to limit our analysis to individuals who were injection drug use–naïve and accessing prescription opioid therapy for noncancer pain, we could not rule out the baseline inclusion of people who inject drugs (owing to imperfect sensitivity of the algorithm or delays in identification) and/or the inclusion of people accessing prescription opioid therapy for recreational purposes or off-label treatment of opioid use disorder. If people who inject drugs were included at baseline, prescription opioid therapy may be a proxy for inadequate access to harm-reduction supplies and/or the high prevalence of chronic pain in this population.^42,43^ In addition, owing to the inability to identify the exact date of seroconversion, some individuals who initiated prescription opioid therapy during follow-up may have done so after acquiring HCV; thus, prescription opioid therapy could be a proxy for HCV-related pain.^44^

Our findings may support the offer of an HCV test to people with a history of long-term prescription opioid therapy^25,26,45^ and may suggest interventions to reduce the risk of prescription opioid dependence (eg, more appropriate and informed opioid prescribing and increased access to multidisciplinary pain management programs)^36,37^ could prevent injection drug use initiation and HCV acquisition. However, interventions to promote appropriate prescription opioid use should consider that unmanaged pain or withdrawal (eg, due to abrupt tapering/discontinuation of therapy) could increase the risk of harm and may facilitate illicit drug use in some individuals.^20,42^ There is a need to support shared, patient-centered decision-making with regard to initiating, tapering, and discontinuing prescription opioid therapy.^46^ Provision of opioid agonist therapy and other harm-reduction interventions to individuals who develop prescription opioid dependence and/or transition to injection drug use can prevent further harms. A systematic review published in 2011 found that harm-reduction strategies combining opioid agonist therapy, sterile drug use equipment, and other supports can reduce HCV incidence by 75% among people who inject drugs.^47^ These interventions should be acceptable, accessible, and affordable and developed through peer engagement.^48^ Provision of prescription opioids as an alternative to the increasingly toxic drug supply may also reduce overdose risk in individuals who initiate use of illicit drugs.^49^

### Limitations

Our study has limitations. Drawbacks of restricting our analysis to individuals who had more than 1 test include exclusion of people whose first HCV test was positive, inability to include all people who were dispensed long-term prescription opioid therapy, and a bias toward including people who transitioned to injection drug use after their HCV-negative test and an elevated rate of HCV seroconversion, limiting generalizability of the study findings. However, given the asymptomatic nature of HCV infection and the lack of a test to define acute infections, the approach we used is necessary to identify seroconversions. For cautious comparison, the annual rate of HCV diagnosis in British Columbia between 2003 and 2015 ranged from 0.5 to 0.8 per 1000 population vs 0.9 per 1000 person-years in our analysis.^50^ Although most HCV infections were likely acquired through injection drug use, some may have been acquired through noninjection drug use routes (eg, sharing of pipes used to smoke illicit drugs, sex, and tattooing).^51,52^ In addition, some evidence suggests certain prescription opioids may suppress the immune system, thereby increasing an individual’s vulnerability to infectious diseases.^53^ We censored individuals who were prescription opioid–naive/acute-exposed who developed a substance use problem between baseline and initiation of long-term prescription opioid therapy to limit the analysis to those who were injection drug use–naive at initiation of therapy, potentially introducing selection bias. We also lacked information on the reason for HCV testing, making it difficult to fully characterize our study population. Our initially injection drug use–naive study population was likely a heterogeneous mix of individuals who received their initial HCV test for different reasons (eg, pregnancy or baby boomers [born between 1945 and 1965]).

## Conclusions

Our large study of persons who received more than 1 HCV test identified an association between medically dispensed long-term prescription opioid therapy for noncancer pain and a higher risk of HCV seroconversion among individuals who were initially injection drug use–naive (1.1% of individuals exposed to long-term prescription opioid therapy seroconverted within 5 years). This finding suggests injection drug use initiation is more frequent among people who received long-term prescription opioid therapy. Our findings may be useful for informing HCV testing and prevention initiatives. However, abrupt tapering or discontinuation of prescription opioid therapy could increase the risk of harm.

## Notes

Supplement. **eTable 1.** Criteria and Data Sources for the BC Hepatitis Testers Cohort (BC-HTC)
eReferences
**eTable 2.** Definitions of Variables Used in the Analysis **eTable 3.** Long-term Episode Characteristics by Episode Type **eTable 4.** Characteristics Associated With HCV Seroconversion in a Cox Multivariable Model
