# Supplementary material for: Association Between Prescription Opioid Therapy for Noncancer Pain and Hepatitis C Virus Seroconversion
Source: JAMA Netw Open. 2022 Jan 12;5(1):e2143050. doi: 10.1001/jamanetworkopen.2021.43050 (PMC8756332; doi:10.1001/jamanetworkopen.2021.43050)
Supplement: Supplement. — eTable 1. Criteria and Data Sources for the BC Hepatitis Testers Cohort (BC-HTC) eReferences eTable 2. Definitions of Variables Used in the Analysis eTable 3. Long-term Episode Characteristics by Episode Type eTable 4. Characteristics Associated With HCV Seroconversion in a Cox Multivariable Model [file jamanetwopen-e2143050-s001.pdf]

Supplementary Online Content

Wilton J, Wong S, Purssell R, et al. Association between prescription opioid therapy for noncancer pain and hepatitis C virus seroconversion. *JAMA Netw Open*. 2022;5(1):e2143050. doi:10.1001/jamanetworkopen.2021.43050

eTable 1. Criteria and Data Sources for the BC Hepatitis Testers Cohort (BC-HTC)

eReferences

eTable 2. Definitions of Variables Used in the Analysis

eTable 3. Long-term Episode Characteristics by Episode Type

eTable 4. Characteristics Associated With HCV Seroconversion in a Cox Multivariable Model

This supplementary material has been provided by the authors to give readers additional information about their work.

eTable 1.Criteria and Data Sources for the BC Hepatitis Testers Cohort (BC-HTC)
Web: https://bchtc.med.ubc.ca/

**Criteria for Inclusion in BC-HTC**	
All individuals:<br>• tested at the BCCDC Public Health Laboratory (BC-PHL) for HCV or HIV OR<br>• reported to BC public health as a confirmed case of HCV, HIV or AIDS, HBV, or active TB OR<br>• included in BC Enhanced Strain Surveillance System (EHSSS) as an acute HBV or HCV case	
All individuals meeting at least one the above criteria were linked internally across all their tests and case reports. Those with a valid personal health number (PHN) were then sent for deterministic linkage with the province-wide Cancer and Ministry of Health (MoH) datasets.	
**Provincial Communicable Disease Data Sources:**	**Data Date Ranges:**
BC-PHL HIV laboratory testing datasets (tests: ELISA, Western blot, NAAT, p24, culture)	1988–2015
BC-PHL HCV laboratory tests datasets (tests: antibody, HCV RNA, genotyping)	1992–2020, Oct
HIV/AIDS Information System (HAISYS) (public health HIV/AIDS case reports)	1980–2015
Integrated Public Health information System (iPHIS) (public health case reports of HCV, HBV, and TB)	1990–2015
Enhanced Strain Surveillance System (EHSSS) (risk factor data on a subset of acute HCV and acute HBV cases)	2000–2013
**Cancer and MoH Administrative Data Sources:**	**Data Date Ranges:**
BC Cancer Registry (BCCR) (primary tumour registry, excludes metastatic cancers)	1923–2016
Client Roster (CR) (Registry of enrollment in the universal public health insurance plan including residential history) [S1]	1990–2016
Discharge Abstracts Dataset (DAD) (hospitalization records) [S2]	1985–2015
Medical Services Plan (MSP) (physician diagnostic and billing data) [S3]	1990–2015
PharmaCare/PharmaNet (Pharma) (prescription drug dispensations) [S4, S5]	1985–2020, Jun
BC Vital Statistics (VS) (deaths registry)[S6]	1985–2020, Jun
NACRS (Emergency Departments) [S7]	2012–2015
Chronic Disease Registry[S8]	1992–2015
The final BC-HTC comprises all individuals successfully linked on PHN to the MoH Client Roster[S1] (a registry of all BC residents enrolled in the publicly-funded universal healthcare system)	

HCV: Hepatitis C Virus; HBV: Hepatitis B Virus; HIV/AIDS: Human Immunodeficiency Virus/Acquired Immunodeficiency Syndrome; BC-PHL: BC Public Health Laboratory: RNA: Ribonucleic Acid; PCR: Polymerase Chain Reaction.

© 2022 Wilton J et al. *JAMA Network Open.*

eReferences

S1. British Columbia Ministry of Health [creator]. Client Roster (Client Registry System/Enterprise Master Patient Index). British Columbia Ministry of Health [publisher]. Data Extract. MOH (2013). 2016. https://www2.gov.bc.ca/gov/content/health/health-forms/online-services

S2. British Columbia Ministry of Health [creator]. Discharge Abstract Database (Hospital Separations). British Columbia Ministry of Health [publisher]. Data Extract. MOH (2013). 2016. https://www2.gov.bc.ca/gov/content/health/health-forms/online-services

S3. British Columbia Ministry of Health [creator]. Medical Services Plan (MSP) Payment Information File. British Columbia Ministry of Health [publisher]. Data Extract. MOH (2013). 2016.https://www2.gov.bc.ca/gov/content/health/health-forms/online-services

S4. British Columbia Ministry of Health [creator]. PharmaCare. British Columbia Ministry of Health [publisher]. Data Extract. MOH (2013). 2020. https://www2.gov.bc.ca/gov/content/health/health-forms/online-services

S5. British Columbia Ministry of Health [creator]. PharmaNet. British Columbia Ministry of Health [publisher]. Data Extract. MOH (2013). 2020. https://www2.gov.bc.ca/gov/content/health/health-forms/online-services

S6. BC Vital Statistics Agency [creator]. Vital Statistics Deaths. BC Vital Statistics Agency [publisher]. Data Extract. BC Vital Statistics Agency (2014). 2020. https://www2.gov.bc.ca/gov/content/health/health-forms/online-services

S7. British Columbia Ministry of Health [creator]. National Ambulatory Care Reporting System. British Columbia Ministry of Health [publisher]. Data Extract. MOH (2017). 2017 https://www2.gov.bc.ca/gov/content/health/health-forms/online-services

S8. British Columbia Ministry of Health [creator]. Chronic Disease Registry. British Columbia Ministry of Health [publisher]. Data Extract. MOH. (2017) 2017. https://www2.gov.bc.ca/gov/content/health/health-forms/online-services

© 2022 Wilton J et al. *JAMA Network Open.*

eTable 2. Definitions of variables used in the analysis

**Substance use problem**<br><br>Defined as first occurrence of either 1 MSP, 1 DAD OR 1 NACRS diagnostic code related to drug psychoses, drug dependence, drug poisoning, nondependent abuse of drugs, drug dependence of mothers (antepartum and postpartum condition or complication), or counselling on substance abuse; OR use of OAT (see below for definition of OAT). Diagnostic codes include injectable and non-injectable drugs (excluding alcohol). Limited to individuals who were age 11-65 years (inclusive) at diagnosis.<br><br>***Physician Billing Data:*** MSP/ICD-9 diagnostic codes starting with 292, 304, 970, 981, 3052, 3053, 3054, 3055, 3056, 3057, 3058, 3059, 6483, 7960, 9621, 9650, 9658, 9663, 9664, 9670, 9671, 9672, 9674, 9675, 9676, 9678, 9679, 9684, 9685, 9694, 9695, 9696, 9697, 9698, 9699, 9710, 9820, 9821, 9823, 9824, 9828, 9872, 9874, 9878, 9892, V5789, E8500 or exact codes 65550, 65551, 65553, V6542.<br><br>***Hospitalization/Emergency Department Data:*** DAD1/DAD3/ICD-9-CM diagnostic codes starting with 292, 304, 970, 981, 3052, 3053, 3054, 3055, 3056, 3057, 3058, 3059, 6483, 7960, 9621, 9650, 9658, 9663, 9664, 9670, 9671, 9672, 9674, 9675, 9676, 9678, 9679, 9684, 9685, 9694, 9695, 9696, 9697, 9698, 9699, 9710, 9820, 9821, 9823, 9824, 9828, 9872, 9874, 9878, 9892, V5789, E8500 or exact codes 65550, 65551, 65553, V6542. DAD2/NACRS/ICD-10-CA diagnostic codes starting with F11, F12, F13, F14, F15, F16, F18, F19, T40, T52, T53, Z503, Z715 or exact codes O35501, O35503, O35509, P044, R781, R782, R783, R784, R785, T387, T412, T423, T424, T425, T426, T427, T428, T436, T438, T439, T507, T590, T598, X41, X42, X62, Y12, Z040. NACRS/ICD-10-CA complaint codes starting with 751, 753.<br><br>*In a validation study using the IDEAs cohort and linked surveillance data with self-reported IDU, this algorithm (in combination with OAT, see below for definition) had a sensitivity of 91% and specificity of 72% for identifying people who inject drugs.*<br><br>**Reference**<br>Janjua NZ, Islam N, Kuo M, Yu A, Wong S, Butt ZA, et al. Identifying injection drug use and estimating population size of people who inject drugs using healthcare administrative datasets. Int J Drug Policy. 2018;55:31–9.
**Opioid agonist therapy (OAT)**<br><br>Defined at occurrence of either 1 MSP fee item code for methadone, buprenorphine, or naloxone treatment OR 1 PharmaNet dispensation for OAT treatment.<br><br>***Physician Billing Data:*** MSP fee item exact code 39.<br><br>***PharmaNet DIN/PINs:*** exact codes: 999776, 999792, 999793, 2241377, 2242963, 2242964, 2295695, 2295709, 2408090, 2408104, 2424851, 2424878, 2453908, 2453916, 2468085, 2468093, 2474921, 2481979, 2483092, 2495872, 2495880, 22123346, 22123347, 22123348, 22123349, 22123357, 66999990, 66999991, 66999992, 66999993, 66999997, 66999998, 66999999, 67000000, 67000001, 67000002, 67000003, 67000004, 67000005, 67000006, 67000007, 67000008, 67000009, 67000010, 67000011, 67000012, 67000013, 67000014, 67000015, 67000016, 67000017, 67000018, 67000019, 67000020.<br><br>*Note: For eligibility criteria, we also excluded those with a methadone or buprenorphine for pain dispensation.*
**Alcohol use problem**<br><br>Defined at the first occurrence of either 2 MSP, 1 DAD OR 1 NACRS diagnostic code for major alcohol-related diagnoses including alcoholic mental disorders and dependence/abuse syndromes; alcoholic

© 2022 Wilton J et al. *JAMA Network Open.*

polyneuropathy, myopathy, cardiomyopathy; pseudo Cushing's syndrome; or discharge to alcohol rehabilitation, counselling, or surveillance.<br><br>***Physician Billing Data:*** MSP/ICD-9 diagnostic codes starting with 291, 303, 3050, 3575, 4255<br>***Hospitalization/Emergency Department Data:*** DAD1/DAD3/ICD-9-CM diagnostic codes starting with 291, 303, 3050, 3575, 4255; DAD2/NACRS/ICD-10-CA diagnostic codes starting with F10, E244, G312, G621, G721, I426, Z502, Z714
**Major mental health illness**<br><br>Major mental illness was defined at the first occurrence of either 2 MSP, 1 DAD OR 1 NACRS code from a psychiatrist visit for schizophrenic, bipolar, delusional, nonorganic psychotic, adjustment, anxiety, dissociative, personality and major depressive disorders.<br><br>***Physician Billing Data:*** MSP claim specialty = 3 AND ICD-9 diagnostic codes starting with 295-298, 300-301, 308-309, 311 or exact code for 50B.<br>***Hospitalization/Emergency Department Data:*** DAD1/ICD-9-CM diagnostic codes starting with 295-298, 300-301, 308-309, 311; DAD2/ICD-10-CA diagnostic codes starting with F20-F25, F28-F34, F38-F45, F48, F60-F61
**Chronic pain**<br><br>Chronic pain was defined at the first occurrence of either 1 MSP or 1 DAD for a 'highly likely' pain diagnostic code OR first occurrence of 2 MSP or 2 DAD (at least 30 days apart) for a 'likely' pain diagnostic code for a range of primarily joint and musculoskeletal disorders (spondylitis, arthritis, spinal stenosis, fibromyalgia).<br><br>**i) 'Highly likely' codes**<br>***Physician Billing Data:*** MSP/ICD-9 diagnostic codes starting with 3382, 3384; MSP fee-item = 1016, 1116 and 1107.<br>***Hospitalization Data:*** DAD1/ICD-9-CM diagnostic codes starting with 3382, 3384. DAD2/ICD-10-CA starting with R521, R522.<br><br>**ii) 'Likely' codes**<br>***Physician Billing Data:*** MSP/ICD-9 diagnostic codes starting with 30780, 30789, 3380, 71941, 71945, 71946, 71947, 71949, 720, 721, 722, 723, 724, 7290, 7291, 7292, 7294, 7295 (but excluded if starting with 7201,7208, 7215, 7217, 7232, 72403, 72471)<br>***Hospitalization Data:*** DAD1/ICD-9-CM diagnostic codes starting with 30780, 30789, 3380, 71941, 71945, 71946, 71947, 71949, 720, 721, 722, 723, 724, 7290, 7291, 7292, 7294, 7295 (but excluded if starting with 7201,7208, 7215, 7217, 7232, 72403, 72471). DAD2/ICD-10-CA starting with F454, M081, M2550, M2551, M2555, M2556, M2557, M432, M433, M434, M435, M436, M45, M461, M463, M464, M469, M47, M480, M481, M488, M489, M500, M502, M503, M508, M509, M51, M531, M532, M533, M538, M539, M54, M608, M609, M633, M725, M790, M791, M792, M796, M797, M961.<br><br>**References:**<br>1) Tian TY, Zlateva I, Anderson DR. Using electronic health records data to identify patients with chronic pain in a primary care setting. J Am Med Inform Assoc. 2013 Dec 1;20(e2):e275–e280.<br>2) Tonelli M, Wiebe N, Fortin M, Guthrie B, Hemmelgarn BR, et al. Methods for identifying 30 chronic conditions: application to administrative data. BMC Med Inform Decis Mak.
**Human immunodeficiency virus (HIV) infection**<br><br>HIV coinfection was defined at the first occurrence of 3 MSP or 1 DAD code for HIV, or a positive HIV serologic test, HAISYS or BC Vital Statistics indication.

**Physician Billing Data:** MSP/ICD-9 diagnostic codes starting with 042, 043, or 044 or exact for 7953, 7958, 79571 or V08.<br>**Hospitalization Data:** DAD1/ICD-9-CM diagnostic codes starting with 042, 043 or 044 or exact code for 7953, 7958, 79571 or V08; DAD2/ICD-10-CA diagnostic codes starting with B20-B24, B9735, F024, O987, R75, Z21.
**Material and Social Deprivation Quintiles**<br>The Québec Index of Material and Social Deprivation was calculated based on individuals' 6-digit postal code. The deprivation index combines six indicators related to health and welfare that represent material or social deprivation and are available by enumeration area in Canadian census data: 1) proportion of persons without high-school diploma 2) ratio of employment to population 3) average income 4) proportion of persons separated, divorced, widowed 5) the proportion of single-parent families 6) proportion of people living alone.<br><br>**Reference:** Pampalon R, Gamache P, Hamel D. A deprivation index for health planning in Canada. Chronic Diseases in Canada 2009; 29.
**Ethnicity**<br>Ethnicity was determined using a validated name recognition algorithm, Onomap[1,2], and classified as South Asian (Pakistani, Indian, Bangladeshi, Nepalese, Sri Lankan), East Asian (Chinese, Japanese, Korean, Filipino, Southeast Asian) or Other residents of BC.<br>In an internal validation study, Onomap had high specificity for South Asian (98.6%) and East Asian (99.5%); the sensitivity was high for South Asian (93.0%), but lower for East Asian (66.7%).<br>**References**<br>1) Ryan R, Vernon S, Lawrence G, Wilson S. Use of name recognition software, census data and multiple imputation to predict missing data on ethnicity: application to cancer registry records. BMC Med Inform Decis Mak. 2012;12:3. doi:10.1186/1472-6947-12-3<br>2) Lakha F, Gorman DR, Mateos P. Name analysis to classify populations by ethnicity in public health: Validation of Onomap in Scotland. Public Health. 2011;125(10):688-696. doi:10.1016/J.PUHE.2011.05.003

BCCDC-PHL, British Columbia Centre for Disease Control Public Health Laboratory; DIN, Drug Identification Number; PIN, Product Identification Number; ICD, International Classification of Diseases; MSP, Medical Services Plan (physician billing data); DAD, Discharge Abstract Database (hospitalization data).

© 2022 Wilton J et al. *JAMA Network Open.*

eTable 3. Long-term episode characteristics by episode type

	All long-term (N=41,755)	Episodic (N=34,681)	Chronic (N=7,074)
Length (days) – median (IQR)	228 (148-664)	191 (139-385)	1,968 (658-3,999)
Days' supply – median (IQR)	40 (15-186)	28 (13-73)	1,389 (439-2,884)
Episode intensity (%) – median (IQR)	17.1 (8.6-38.3)	13.5 (7.6-24.7)	73.0 (61.3-84.8)
Average daily dose (MEQ) – median (IQR)	22.5 (14.7-36.0)	21.2 (14.3-32.1)	35.1 (18.0-74.2)
PO formulation			
Codeine IR only (%)	47.8	54.2	16.8
Any higher potency PO dispensation (hydromorphone, morphine, oxycodone, fentanyl) (%)	39.8	32.9	73.4
Any ER formulation (%)	15.9	7.5	57.2

IR, immediate release; ER, extended release; IQR, interquartile range; PO, prescription opioid; MEQ, morphine equivalents.

© 2022 Wilton J et al. *JAMA Network Open.*

eTable 4. Characteristics associated with HCV seroconversion in a Cox multivariable model.

	aHR (95%CI)
Long-term PO exposure (vs. PO-naïve/acute)	3.2 (2.9-3.6)
Sex (male vs. female)	1.9 (1.8-2.1)
Ethnicity (vs. Other residents of BC)	
East Asian	0.2 (0.2-0.3)
South Asian	0.4 (0.3-0.5)
Material deprivation (vs. 1, least)	
2	1.3 (1.1-1.6)
3	1.6 (1.3-1.9)
4	2.3 (2.0-2.7)
5 (most)	3.0 (2.6-3.6)
Missing	2.2 (1.4-3.4)
Chronic pain (yes vs. no)	0.7 (0.6-0.7)
Major mental illness (yes vs. no)	1.0 (0.9-1.2)

PO, prescription opioid; BC, British Columbia; aHR, adjusted hazard ratio; CI, confidence interval. Models were stratified by calendar year (2000-2003, 2004-2008, 2009-2015) and age (<25, 25-44, 45-54, 55+) due to violation of non-proportionality assumption. Model also adjusted for geography (health authority). 'Missing' material deprivation retained in model as may be a proxy for homelessness.

© 2022 Wilton J et al. *JAMA Network Open.*
